# A Broader View: Microbial Enzymes and Their Relevance in Industries, Medicine, and Beyond

**DOI:** 10.1155/2013/329121

**Published:** 2013-09-11

**Authors:** Neelam Gurung, Sumanta Ray, Sutapa Bose, Vivek Rai

**Affiliations:** ^1^Department of Earth Sciences, Indian Institute of Science Education and Research-Kolkata Nadia, Mohanpur, West Bengal 741252, India; ^2^Institute of Life Sciences, Bhubaneswar 751023, India

## Abstract

Enzymes are the large biomolecules that are required for the numerous chemical interconversions that sustain life. They accelerate all the metabolic processes in the body and carry out a specific task. Enzymes are highly efficient, which can increase reaction rates by 100 million to 10 billion times faster than any normal chemical reaction. Due to development in recombinant technology and protein engineering, enzymes have evolved as an important molecule that has been widely used in different industrial and therapeutical purposes. Microbial enzymes are currently acquiring much attention with rapid development of enzyme technology. Microbial enzymes are preferred due to their economic feasibility, high yields, consistency, ease of product modification and optimization, regular supply due to absence of seasonal fluctuations, rapid growth of microbes on inexpensive media, stability, and greater catalytic activity. Microbial enzymes play a major role in the diagnosis, treatment, biochemical investigation, and monitoring of various dreaded diseases. Amylase and lipase are two very important enzymes that have been vastly studied and have great importance in different industries and therapeutic industry. In this review, an approach has been made to highlight the importance of different enzymes with special emphasis on amylase and lipase in the different industrial and medical fields.

## 1. Introduction

Enzymes are the biological substance or biological macromolecules that are produced by a living organism which acts as a catalyst to bring about a specific biochemical reaction. These are like the chemical catalysts in a chemical reaction which helps to accelerate the biological/biochemical reactions inside as well as outside the cell. These are generally known as “Biocatalyst.” In 1877, Wilhelm Friedrich Kühne a professor of physiology at the University of Heidelberg first used the term enzyme, which comes from a Greek word *ενζυμον* meaning “in leaven" [1]. Even many centuries ago enzyme and its use were well known to the mankind but Wilhelm Friedrich Kühne was the first person to give a scientific terminology to this biomolecule. Use of enzyme has been seen in ancient Egyptians where they were used for the preservation of food and beverages. Cheese making has always involved the use of enzymes, and it goes as far as back in about 400 BC, when Homer's Iliad mentioned the use of a kid's stomach for making cheese. In 1783, the famous Italian catholic priest Lazzaro Spallanzani first mentioned the importance of this biomolecule in his work of biogenesis (spontaneous generation of microbes) where he mentioned that there is a life-generating force inherent to certain kinds of inorganic matter that causes living microbes to create themselves given sufficient time [2]. In the year 1812 Gottlieb Sigismund Kirchhoff was investigating the procedure of converting starch into glucose. In his experiment he also enlightens the application of these biomolecules as catalyst [3]. In 1833, French chemist Anselme Payen discovered the first enzyme, diastase [4]. In 1835, the hydrolysis of starch by diastase was acknowledged as a catalytic reaction by another Swedish scientist Jöns Jacob Berzelius. In 1839, he also interpreted fermentation as being caused by a catalytic force and postulated that a body—by its mere presence—could, by affinity to the fermentable substance, cause its rearrangement to the products [5]. In 1846 the activity of invertase was demonstrated by Dubonfout. A few decades later in 1862, when studying the fermentation of sugar to alcohol by yeast, Louis Pasteur along with Ferdinand Cohn and Robert Koch came to the conclusion that this fermentation was catalysed by a vital force contained within the yeast cells called “ferments," which were thought to function only within living organisms [6]. In 1894, Jokichi Takamine discovered takadiastase which is the form of diastase obtained from *Aspergillus oryzae*. In 1897, Eduard Buchner demonstrated the conversion of glucose to ethanol by a cell-free extract from the yeast. Later in 1908, Otto Rohm, German scientist, introduced application of pancreatic enzymes with inorganic salts to meet the requirement in tanneries for bating of hides. In 1916, J. M. Nelson and E. G. Griffin showed the adsorption of invertase on charcoal and alumina demonstrating that immobilised enzymes can be retained. At present, immobilized cells have been used for production of organic acids, amino acids, antibiotics, enzymes, alcohol, and other compounds. Immobilized cell techniques have several advantages as compared to the free cell system, such as higher production rate and easier product separation [7]. It was not until 1926, however, that the first enzyme was obtained in pure form, a feat accomplished by James B. Sumner of Cornell University. Sumner in 1947 was able to isolate and crystallize the enzyme urease from the jack bean. His work was to earn him the Nobel Prize. John H. Northrop and Wendell M. Stanley shared the 1947 Nobel Prize with Sumner. They discovered a complex procedure for isolating pepsin. This precipitation technique devised by Northrop and Stanley has been used to crystallize several enzymes [8]. In 1960, NOVO started producing protease using Bacillus lichnformis on a commercial scale. After 1980, many scientists started application of genetic engineering techniques in order to improve the production of enzymes and also to alter the properties of enzymes by protein engineering.

Naturally found enzymes have been used widely since ancient times and in the manufacture of products such as linen, leather, and indigo. All of these processes dependent on either enzymes produced by microorganisms or enzymes present in added preparations such as calves' rumen or papaya fruit. The development of fermentation processes was aimed specifically at the production of enzymes by use of particularly selected strains, due to which it is possible to produce purified, well-characterized enzymes on a large scale. This development allowed the introduction of enzymes into true industrial products and processes, for example, within the detergent, textile, and starch industries. The recombinant DNA technology has further improved production processes and helped to produce enzymes commercially that could not be produced previously. Furthermore, the developments in biotechnology, such as protein engineering and directed evolution, further revolutionized the commercialization of industrial important enzymes. This advance in biotechnology is providing different kinds of enzymes displaying new activities, adaptability to new conditions leading to their increase use in industrial purposes. Since 1940s, the intensive research biochemistry confronted the use of enzymes as diagnostic tool and also provided basis in clinical chemistry. It is, however, only within the recent past few decades that interest in diagnostic enzymology has multiplied. Many methods currently on record in the literature are not in wide use, and there are still large areas of medical research in which the diagnostic potential of enzyme reactions has not been explored at all.

The majority of currently used industrial enzymes are hydrolytic in action, being used for the degradation of various natural substances. Proteases remain the dominant enzyme type, because of their extensive use in the detergent and dairy industries. Various carbohydrases, primarily amylases and cellulases, used in industries such as the starch, textile, detergent, and baking industries, represent the second largest group [9]. The global market for industrial enzymes is estimated at 3.3 billion dollars in 2010. This market is expected to reach more than 4 billion dollars by 2015. Enzymes play key roles in numerous biotechnology products and processes that are commonly encountered in the production of food and beverages, cleaning supplies, clothing, paper products, transportation fuels, pharmaceuticals, and monitoring devices. At present, the most frequently used enzymes in biotechnology are hydrolases, which catalyse the breakdown of molecules. Enzymes can display regional stereospecificity, properties that have been exploited for asymmetric synthesis and racemic resolution. Chiral selectivity of enzymes has been employed to prepare enantiomerically pure pharmaceuticals, agrochemicals, chemical feedstock, and food additives.

Thus, enzymes do show us a wide range of applications in different industries whether it may be food, textile, medicine, dairy, or any other. With the advancement of modern biotechnology and protein engineering we have the capability to introduce or modify the capability of the genes that are important for us to produce these novel enzymes. Our objective in writing this review is to emphasize the current role of the microbial enzymes and the current status of their use in different industries along with the biotechnological perspectives of its future development.

## 2. Enzymes, Classification, and Their Use

Enzymes are large biological molecules responsible for all those important chemical interconversions that are required to sustain life [10]. They are highly selective catalysts which can greatly accelerate both the rate and specificity of metabolic reactions, which range from the digestion of food to the synthesis of DNA. Almost all chemical reactions in a biological cell need enzymes in order to occur at rates sufficient for life. Since enzymes are selective for their substrates and speed up only a few reactions from among many possibilities, the set of enzymes made in a cell determines which metabolic pathways occur in that cell. Enzymes are known to catalyse about 4,000 biochemical reactions [11]. Enzymes are very specific, and it was suggested by the Nobel laureate Emil Fischer in 1894 that this was because both the enzyme and the substrate possess specific complementary geometric shapes that fit exactly into one another [12]. This is often referred to as “the lock and key" model. However, while this model explains enzyme specificity, it fails to explain the stabilization of the transition state that enzymes achieve.

Most enzymes are much larger than the substrates they act on, and only a small portion of the enzyme (around 2–4 amino acids) is directly involved in catalysis [13]. The region that contains these catalytic residues, binds the substrate, and then carries out the reaction is known as the active site. Enzymes can also contain sites that bind cofactors, which are needed for catalysis. Some enzymes also have binding sites for small molecules, which are often direct or indirect products or substrates of the reaction catalyzed. This binding can serve to increase or decrease the enzyme's activity, providing a means for feedback regulation. Like all proteins, enzymes are long, linear chains of amino acids that fold to produce a three-dimensional product. Each unique amino acid sequence produces a specific structure, which has unique properties. Individual protein chains may sometimes group together to form a protein complex. Most enzymes can be denatured that is, unfolded and inactivated by heating or chemical denaturants, which disrupt the three-dimensional structure of the protein. Depending on the enzyme, denaturation may be reversible or irreversible.

Due to their wide range of activities based on their nature of reaction enzymes are being classified according to their enzyme catalysing reaction as shown in Table 1. The Enzyme Commission number (EC number) is a numerical classification scheme for enzymes, based on the chemical reactions they catalyze [14]. As a system of enzyme nomenclature, every EC number is associated with a recommended name for the respective enzyme. Except for some of the originally studied enzymes such as pepsin, rennin, and trypsin, most enzyme names end in “ase." The enzyme nomenclature scheme was developed starting in 1955, when the International Congress of Biochemistry in Brussels sets up an Enzyme Commission. The first version was published in 1961. The current sixth edition, published by the International Union of Biochemistry and Molecular Biology in 1992, contains 3196 different enzymes. The International Union of Biochemistry (I.U.B.) initiated standards of enzyme nomenclature which recommend that enzyme names indicate both the substrate acted upon and the type of reaction catalyzed. According to the enzyme commission the enzymes are divide into 6 parts:

- oxidoreductase (EC 1),
- transferase (EC 2),
- hydrolase (EC 3),
- lyase (EC 4),
- isomerase (EC 5),
- ligase (EC 6).

In Table 2 examples of few classes of industrially important enzymes are given.

## 3. Application of Enzyme

### 3.1. Amylase

**Figure 1:**
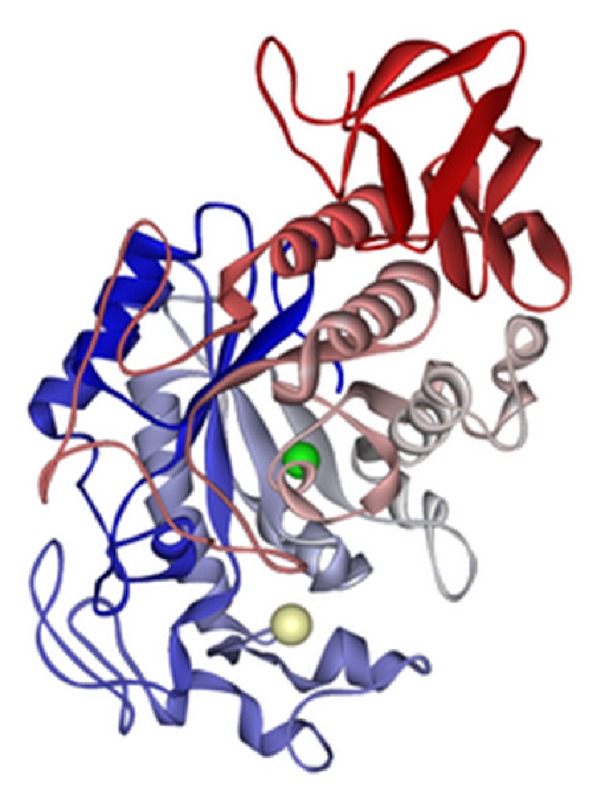
Computer simulated 3D image of human salivary amylase [22].

Amylase is an enzyme that catalyses the breakdown of starch into sugars. Amylase is abundantly present in human saliva as shown in Figure 1, where it begins the mechanical process of digestion. Foods that contain much starch but little sugar, such as rice and potato, taste slightly sweet as they are chewed because amylase turns some of their starch into sugar in the mouth. The pancreas also makes amylase (alpha amylase) to hydrolyse dietary starch into disaccharides and trisaccharides which are converted by other enzymes to glucose to supply the body with energy. Plants and some bacteria also produce amylase. As diastase, amylase was the first enzyme to be discovered and isolated by Anselme Payen in 1833. All amylases are glycoside hydrolases and act on *α*-1,4-glycosidic bonds. It is widely used in industries and have nearly 25% of the enzyme market [15, 16]. Today amylase has almost replaced chemical hydrolysis of starch in starch processing industry. The amylases obtained from microorganisms have a broad spectrum of industrial uses as they are more stable than plant and animal amylases. The major advantage of using microorganisms for the production of amylases is the economical bulk production capacity, and also microbes are easy to manipulate to derive enzymes of desired nature. Starch-degrading amylolytic enzymes are of great importance in biotechnological sector ranging from food, fermentation, textile to paper industries [17, 18]. Although amylases can be obtained from several sources, like plants and animals, the enzymes from microbial sources generally satisfy industrial demands and had made significant contribution to the foods and beverages industry in the last three decades.

### 3.2. Types

Amylase has been divided into three sub classes—*α*- *β*- *γ*-amylase. The classification is based on the bonding type.

### 3.3. *α*-Amylase

*α*-Amylase are enzymes that helps in the hydrolysis of internal *α*-1,4-glycosidic linkages in starch in low molecular weight products, such glucose, maltose, and maltotriose units [19, 20]. It is the major form of amylase found in humans and other mammals [21]. It is also present in seeds containing starch as a food reserve and is secreted by many fungi. Although found in many tissues, amylase is most prominent in pancreatic juice and saliva, each of which has its own isoform of human *α*-amylase. In humans, all amylase isoforms link to chromosome 1p21 (AMY1A).

### 3.4. Use

*α*-Amylase is used in ethanol production to break starches in grains into fermentable sugars. The first step in the production of high-fructose corn syrup is the treatment of cornstarch with *α*-amylase, producing shorter chains of sugars called oligosaccharides. An *α*-amylase called “Termamyl," sourced from *Bacillus licheniformis*, is also used in some detergents, especially dishwashing and starch-removing detergents.

### 3.5. *β*-Amylase

**Figure 2:**
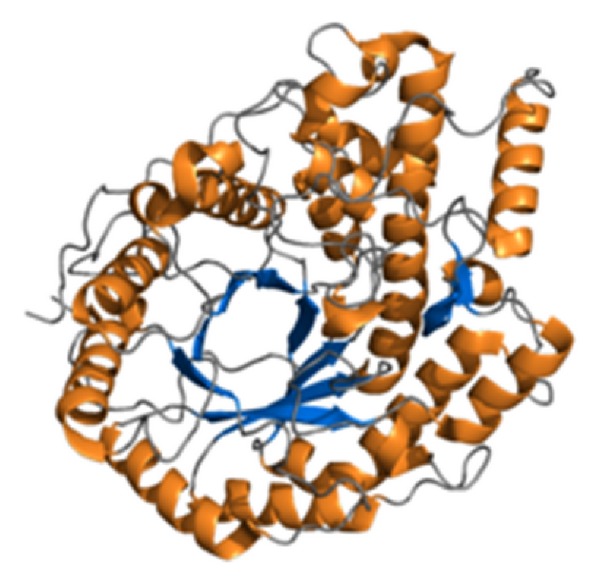
Computer simulated 3D image of barley beta-amylase [23].

Another form of amylase, *β*-amylase (EC 3.2.1.2 ) (alternative names: 1, 4-*α*-D-glucan maltohydrolase; glycogenase; saccharogen amylase) is also synthesized by bacteria, fungi, and plants and shown in Figure 2. Working from the nonreducing end, *β*-amylase catalyzes the hydrolysis of the second *α*-1,4 glycosidic bond, cleaving off two glucose units (maltose) at a time. During the ripening of fruit, *β*-amylase breaks starch into maltose, resulting in the sweet flavor of ripe fruit. Both *α*-amylase and *β*-amylase are present in seeds; *β*-amylase is present in an inactive form prior to germination, whereas *α*-amylase and proteases appear once germination has begun. Cereal grain amylase is key to the production of malt. Many microbes also produce amylase to degrade extracellular starches. Animal tissues do not contain *β*-amylase, although it may be present in microorganisms contained within the digestive tract. The optimum pH for *β*-amylase is 4-5.

### 3.6. Use

*α* and *β* amylases are important in brewing beer and liquor made from sugars derived from starch. In fermentation, yeast ingest sugars and excrete alcohol. In beer and some liquors, the sugars present at the beginning of fermentation have been produced by “mashing" grains or other starch sources (such as potatoes). In traditional beer brewing, malted barley is mixed with hot water to create a “mash," which is held at a given temperature to allow the amylases in the malted grain to convert the barley's starch into sugars. Different temperatures optimize the activity of *α* and *β* amylase, resulting in different mixtures of fermentable and unfermentable sugars. In selecting mash temperature and grain-to-water ratio, a brewer can change the alcohol content, mouthfeel, aroma, and flavor of the finished beer.

### 3.7. *γ*-Amylase

**Figure 3:**
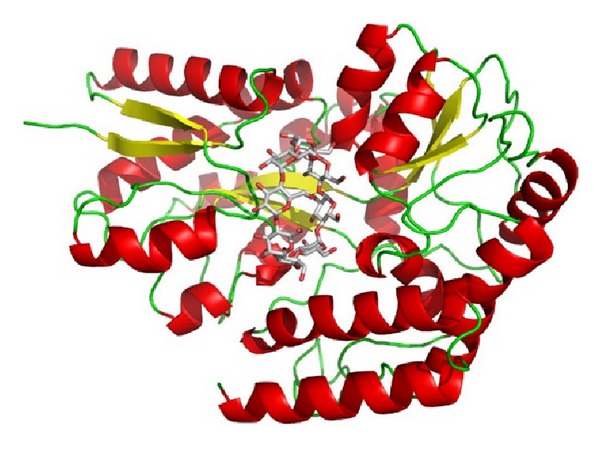
Computer simulated 3D image of gamma amylase from *Thermoactinomyces vulgaris* R-47 cyclodextrin binding protein (2DFZ) [24].

*γ*-Amylase (EC 3.2.1.3 ) (alternative names: Glucan 1,4-*α*-glucosidase; amyloglucosidase; Exo-1,4-*α*-glucosidase; glucoamylase; lysosomal *α*-glucosidase; 1,4-*α*-D-glucan glucohydrolase) will cleave *α*(1-6) glycosidic linkages, as well as the last **α**(1-4) glycosidic linkages at the nonreducing end of amylose and amylopectin, yielding glucose. The image of *γ*-amylase is shown in Figure 3. The *γ*-amylase has most acidic pH optimum because it is most active around pH 3.

### 3.8. Use

They are used in food, pharmaceutical, drug delivery, and chemical industries, as well as agriculture and environmental engineering. Hydroxypropyl beta cyclodextrin (HP*β*CD) is the chief active compound found in Procter and Gamble's deodorizing product “Febreze" under the brand name “Clenzaire."

### 3.9. Bacterial Amylases

Amylase can be obtained from different species of microorganisms, but for commercial use, *α*-amylase derived from *Bacillus licheniformis*, *Bacillus stearothermophilus*, and *Bacillus amyloliquefaciens* has number of application in different industries such as in food, fermentation, textiles and paper industries [25, 26]. Thermostability is a desirable characteristic of a major group of industrial enzymes. Thermostable enzymes have found a large number of commercial applications due to their stability. Thermostable amylolytic enzymes have been currently in research to improve industrial method of starch degradation and also in the production of valuable products like crystalline dextrose, glucose, maltose, dextrose syrup, and maltodextrins [27, 28]. *Bacillus subtilis, Bacillus stearothermophilus, Bacillus licheniformis,* and *Bacillus amyloliquefaciens* are found to be good producers of thermostable *α*-amylase and are widely in use for commercial production of the enzyme for numerous applications. Today, thermostable amylases of *Bacillus stearothermophilus* or *Bacillus licheniformis* are used in starch processing industries [28]. Some halophilic microorganisms have optimal activity at high salinity and enzymes produced by them could be used in many harsh industrial processes where highly concentrated salt solutions are used which would otherwise inhibit many enzymatic reactions [29]. Also, most of the halobacterial enzymes are thermotolerant and remain stable at room temperature for a long period [30]. Halophilic amylases have been derived from bacteria such as *Chromohalobacter *sp. [29], *Halobacillus *sp. [31], *Haloarcula hispanica* [32], *Halomonas meridiana* [33], and *Bacillus dipsosauri* [34].

### 3.10. Fungal Amylase

Most of the mesophilic fungi are reported to produce *α*-amylase, and many researches have been done for specific cultural conditions and to choose the best strains to produce commercially. Fungal enzymes are limited to terrestrial isolates, mostly to *Aspergillus and Penicillium* [35]. The *Aspergillus* species usually produces a variety of extracellular enzymes, and amylases are the ones with the most significant industrial value. Filamentous fungi, such as *Aspergillus oryzae* and *Aspergillus niger*, produce large quantities of enzymes that can be used extensively in the industry. *A. oryzae* is considered to be the favourable host for the production of heterologous proteins as it has ability to secrete a vast amount of high value proteins and industrial enzymes, for example, *α*-amylase [28]. *Aspergillus oryzae* has been extensively used in the production of food such as soy sauce and organic acid such as citric and acetic and commercial enzymes including *α*-amylase [36]. *Aspergillus niger* is acid tolerant (pH < 3) and hence has important hydrolytic capacities in the *α*-amylase production, and it also avoids bacterial contamination [37]. The fungal *α*-amylase is usually preferred over other microbial sources because of their more accepted (GRAS) Generally Recognized as Safe status [19]. The thermophilic fungus *Thermomyces lanuginosus* is an excellent producer of amylase. Jensen et al. [38] and Kunamneni et al. [39] purified the *α*-amylase, proving its thermostability.

### 3.11. Application of Amylase

Amylases have a wide range of application in various industries such as in the food, bread making, paper industries, textiles, sweeteners, glucose and fructose syrups, fruit juices, detergents, fuel ethanol from starches, alcoholic beverages, digestive aid, and spot remover in dry cleaning. Bacterial *α*-amylases are also being used in clinical, medicinal, and analytical chemistry [26]. The widely used thermostable enzymes in the starch industry are the amylases [38].

### 3.12. Use in Starch Industry

The starch industry has the most widespread applications of amylases, which are used during starch hydrolysis in the starch liquefaction process that converts starch into fructose and glucose syrups [40]. The enzymatic conversion of all starch includes gelatinization, which involves the dissolution of starch granules, thereby forming a viscous suspension; liquefaction, which involves partial hydrolysis and loss in viscosity; and saccharification, involving the production of glucose and maltose via further hydrolysis [28].

### 3.13. Use in Detergent Industry

Both in terms of volume and value detergent industry are the primary consumers of enzymes. The application of enzymes in detergents making enhances the detergents ability to remove tough stains and also makes detergent ecofriendly. Amylases are the second type of enzymes used in the detergent formulation, and 90% of all liquid detergents contain these enzymes [41]. These enzymes are used for laundry and automatic dishwashing to clean up residues of starchy foods such as custard, gravies, potato, and chocolate. and other smaller oligosaccharides.

### 3.14. Use in Food Industry

There is an extensive use of amylase in processed food industry such as baking, brewing, production of cakes, preparation of digestive aids, fruit juices, and starch syrups. The *α*-amylases have been used in the baking industry widely [42]. These enzymes are generally added to the dough of bread in order to degrade the starch into smaller dextrins, which are further fermented by the yeast. The *α*-amylase enhances the fermentation rate and the reduction of the viscosity of dough, which results in improvements in the volume and texture of the product.

### 3.15. Use in Textile Industry

Amylases are utilized for desizing process in textile industry. Sizing agents like starch are added to yarn before fabric production for fast and secure weaving process. Starch is a very attractive size, because it is cheap, easily available all over, and it can be easily removed. Desizing is the process where removal of starch from the fabric takes place and acts as the strengthening agent to prevent breaking of the warp thread during the weaving process. The *α*-amylases selectively remove, the size and do not affect the fibres [43]. For a long time amylase from *Bacillus* strain was employed in textile industry.

### 3.16. Use in Paper Industry

The main use of *α*-amylases in the pulp and paper industry is the modification of starch of coated paper, that is, for the production of low-viscosity, high-molecular weight starch [42]. The coating treatment makes the surface of paper smooth and strong to improve the writing quality of the paper. For paper sizing the viscosity of natural enzyme is too high, and this can be changed by partially degrading the polymer with *α*-amylases in a batch or continuous processes. Starch is considered to be the good sizing agent for the finishing of paper, improving the quality and reusability, besides being a good coating for the paper.

### 3.17. Use in Medicine

A higher than normal concentration of amylases may predict one of several medical conditions, including acute inflammation of the pancreas, perforated peptic ulcer, strangulation ileus, torsion of an ovarian cyst, macroamylasemia, and mumps. In other body fluids also amylase can be measured, including urine and peritoneal fluid. In various human body fluids the level *α*- amylase activity is of clinical importance, for example, in diabetes, pancreatitis, and cancer research [44].

### 3.18. Lipase

**Figure 4:**
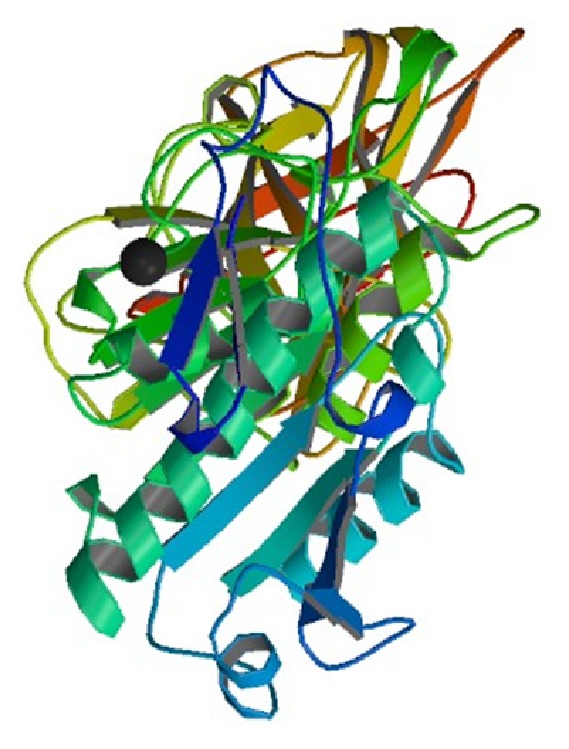
Computer simulated 3D image of pancreatic lipase [48].

It is an enzyme that catalyzes the breakdown or hydrolysis of fats [45]. Lipases are a subclass of the esterases. Lipases perform essential roles in the digestion, transport, and processing of dietary lipids (e.g., triglycerides, fats, and oils) in most, if not all, living organisms. Genes encoding lipases are even present in certain viruses [46]. Most lipases act at a specific position on the glycerol backbone of lipid substrate especially in small intestine. For example, human pancreatic lipase as shown in Figure 4, which is the main enzyme that breaks down dietary fats in the human digestive system, converts triglyceride substrates found in ingested oils to monoglycerides and two fatty acids. Several other types of lipase activities exist in nature, such as phospholipases and sphingomyelinases; however, these are usually treated separately from “conventional" lipases. Some lipases are expressed secreted by pathogenic organisms during the infection. In particular, *Candida albicans* has a large number of different lipases, possibly reflecting broad lipolytic activity, which may contribute to the persistence and virulence of *C. albicans* in human tissue [47]. Lipases are considered as major group of biotechnologically valuable enzymes, mainly due to the versatility of their applied properties and easy mass production. Microbial lipases are largely diversified in their enzymatic properties and substrate specificity, which make them potential source for industrial applications. The interest in this enzyme is primarily due to investigations of their role in pathogenesis and their wide application in biotechnology. Bacterial lipases are more stable than animal or plant lipases. The energy expenditure required to conduct reactions at elevated temperatures and pressures is eliminated as lipases are active under ambient temperature, and it also reduces the denaturation of labile reactants and products.

### 3.19. Types

There are no such distinguished types of lipase, but mainly it is categorized according to its use, namely, human digestive system in human pancreatic lipase (HPL) and pancreatic lipase. Others include hepatic lipase (HL), endothelial lipase, and lipoprotein lipase.

### 3.20. Application of Lipase

Lipases are involved in diverse biological processes ranging from routine metabolism of dietary triglycerides to cell signaling and inflammation [53, 54]. Thus, some lipase activities are confined to specific compartments within cells, while others work in extracellular spaces.

- In the example of lysosomal lipase, the enzyme is confined within an organelle called the lysosome.
- Other lipase enzymes, such as pancreatic lipases, are secreted into extracellular spaces where they serve to process dietary lipids into more simple forms that can be more easily absorbed and transported throughout the body.
- Fungi and bacteria may secrete lipases to facilitate nutrient absorption from the external medium (or in examples of pathogenic microbes to promote invasion of a new host).
- Certain wasp and bee venoms contain phospholipases that enhance the “biological payload" of injury and inflammation delivered by a sting.
- As biological membranes are integral to living cells and are largely composed of phospholipids, lipases play important roles in cell biology.
- *Malassezia globosa*, a fungus that is thought to be the cause of human dandruff, uses lipase to break down sebum into oleic acid and increase skin cell production, causing dandruff.

Lipases serve important roles in human practices as ancient as yogurt and cheese fermentation. However, lipases are also being exploited as cheap and versatile catalysts to degrade lipids in more modern applications. For instance, a biotechnology company has brought recombinant lipase enzymes to market for use in applications such as baking, laundry detergents, and even as biocatalysts [55] in alternative energy strategies to convert vegetable oil into fuel [56]. High enzyme activity lipase can replace traditional catalyst in processing biodiesel; this enzyme is more environmental and safe.

### 3.21. Bacterial Lipase

Some of the lipase-producing bacterial genera include *Bacillus*, *Pseudomonas, and Burkholderia*. The commercially important bacterial lipases are usually extracellular, and also their bulk production is much easier. There are a number of lipase-producing bacteria, but only a few are commercially exploited as wild or recombinant strains [57]. Of these, the important ones are *Achromobacter*, *Alcaligenes*, *Arthrobacter*, *Bacillus*, *Burkholderia*, *Chromobacterium*, *Enterococcus*, *Corynebacterium,* and *Pseudomonas*. In a variety of biotechnological applications lipases from *Pseudomonas *are widely used [58, 59]. Various products based on bacterial lipases have been launched in the market in the past few years, such as Lumafast and Lipomax from *Pseudomonas *with their major application in detergent enzymes, while Chiro CLEC-PC, Chirazyme L-1, and Amano P, P-30 and PS have numerous applications in organic synthesis.

### 3.22. Fungal Lipase

Fungi capable of synthesizing lipases are found in several habitats, including soils contaminated with wastes of vegetable oils, dairy byproduct, seeds, and deteriorated food [60, 61]. *Candida rugosa* lipases have been known for their diverse biotechnological potential [62]. The presence of *C. rugosa* lipase isoforms has been reported by many researchers [62, 63]. *Rhizopus oryzae* lipase, *Rhizopus delemar* lipase and *Rhizopus javanicus* lipase have a substitution in the His 134 and the Leu 234 was referred by Minning et al. [64]. The LIP2 lipase from the *Yarrowia lipolytica* (YLLIP2) have a high potential for enzyme replacement therapy due to its unique biochemical properties: It shows highest activity at low pH values and is not repressed by bile salts. It was also reported that *Thermomyces lanuginosus* lipase has variety of applications in the field of detergents and biotechnological processes and it was also reported that YLLIP2 belongs to same family [65]. Other major lipase-producing fungi are *Mucor, Candida, Penicillium, Rhizopus, Geotrichum, Rhizomucor*, *Aspergillus, Humicola, *and *Rhizopus *[66]. The extracellular thermostable lipase is produced by thermophilic *Mucor pusillus*, *Rhizopus homothallicus*, and *Aspergillus terreu*s. *Mucor* sp. produces an extracellular, thermostable, inducible, and alkaliphilic lipase. There are few reports that have been made so far with molds with alkaliphilic and thermostable lipase [67, 68].

### 3.23. Use in Textile Industry

In the textile industry lipases are used for the removal of size lubricants, which increases fabrics absorbance ability for improved levelness in dyeing. In the denim abrasion systems, it is used to lessen the frequency of cracks and streaks. Commercial preparations used for the desizing of denim and other cotton fabrics contain both alpha amylases, and lipase enzymes are used for the desizing of cotton fabrics and denim during its commercial preparation [69].

### 3.24. Use in Detergent Industry

The hydrolytic lipases are commercially very important, and their addition to detergents is mainly used in laundries and household dishwashers. Enzymes reduce the environmental load of detergent products, as they save energy by enabling a lower wash temperature to be used, and use of chemicals in detergents is reduced, mostly biodegradable, leaving no harmful residues has no negative impact on sewage treatment processes; and does not possess any kind of risk to aquatic life [70].

### 3.25. Use in Food Industry

To modify the food flavour by synthesis of esters of short-chain fatty acids and alcohols (flavour and fragrance) lipases have been frequently used. Lipases play a major role in the fermentative steps during manufacturing of sausage and also to measure changes in long-chain fatty acid liberated during ripening. Previously, lipases of different microbial sources were used for refining rice flavour, modifying soybean milk, and for enhancing the aroma and speed up the fermentation of apple wine [71]. By adding lipases the fat is removed while processing meat and fish, and this process is called biolipolysis.

### 3.26. Use in Diagnosis

Lipases are considered as important drug targets or marker enzymes in the medical field. The presence or high levels of lipases can indicate certain infection or disease and can be used as diagnostic tool. They are used in the determination of serum triglycerides to liberate glycerol which is determined by enzyme-linked colorimetric reactions. Acute pancreatitis and pancreatic injury can be determined by the level of lipases in blood [72]. Few new developments have been made by using lipases for the diagnosis of pancreatitis. The development of a test for measurement of canine pancreatic lipase has been developed using pancreatic lipases as they are fixed markers for the pancreas. A serum feline pancreatic lipase immunoreactivity (fPLI) test was currently developed, and findings suggest that this test is more accurate than other diagnostic tools used for the diagnosis of feline pancreatitis [73]. *P. aeruginosa *strains are isolated from patients with cancer, and 69% of the strains showed higher level of lipase (20–150 U/mL); these elevated levels of lipases were involved mainly with nontypable strains. The nontypable strains clearly show the most frequent group with elevated level of lipase, proteinase, elastase, hydrophobicity, and motility [74].

### 3.27. Use in Medical Applications

Lipases isolated from *Galleria mellonella* (wax moth) were found to have a bactericidal action on *Mycobacterium tuberculosis *(MBT) H37Rv. This preliminary research may be considered as part of global unselected screening of biological and other samples for detecting new promising sources of drugs [75]. Lipases can be used as digestive aids. Lipases can be used in the treatment of malignant tumors as they are the activators of tumor necrosis factor. Human gastric lipase (HGL) is the most stable acid lipase and considered to be a good tool for enzyme substitution therapy. Earlier lipases have been used in the treatment of gastrointestinal disturbances, dyspepsias, cutaneous manifestations of digestive allergies, and so forth. Lipase from *Candida rugosa *synthesizes lovastatin, a drug that lowers serum cholesterol level. The asymmetric hydrolysis of 3-phenylglycidic acid ester which is a key intermediate in the synthesis of diltiazem hydrochloride is a widely used coronary vasodilator and is synthesized using *S. marcescens *lipase [76].

### 3.28. Use in Cosmetics

Retinoids (vitamin A and derivatives) are commercially very important in cosmetics and pharmaceuticals such as skin care products. Immobilized lipases are used for the preparation of water-soluble retinol derivatives. Lipases are used in hair waving preparation and have also been used as an ingredients of topical antiobese creams or as oral administration [77].

### 3.29. Use as Biosensor

The enzyme-catalysed dissolution of biodegradable polymer films based on biosensor has been developed. The polymer enzyme system. poly(trimethylene) succinate, Which was investigated is degraded by a lipase and can be used as biosensor. Within the last few years, different processes have been designed using enzyme-labelled probes in order to avoid unstable and harmful isotopes. While screening various hydrolytic enzymes to fulfil the special demands, fungal lipases turned out to be the most relevant one. Immobilization of lipases can be done on pH/oxygen electrodes along with glucose oxidase, and these serve as lipid biosensors and can be used in triglycerides and blood cholesterol determinations [78].

### 3.30. Use in Biodegradation

Margesin et al. [79] have concluded that soil microbial lipase activity can be an important indicator of diesel oil biodegradation in freshly contaminated, unfertilized, and fertilized soils. In the coastal environment fungal strains are used to degrade oil spills, which in turn increase ecorestoration and enzymatic oil processing in industries. Lipase produced by *Bacillus subtilis, Bacillus licheniformis, Bacillus amyloliquefaciens, Serratia marcescens, Pseudomonas aeruginosa, *and *Staphylococcus aureus *was reported to degrade palm oil mill, diary, slaughter house, soap industry, and domestic waste water [80]. *Pseudomonas aeruginosa* lipases were recommended for castor oil degradation [81].

## 4. General Therapeutic Application of Other Enzymes

Therapeutic enzymes have a wide variety of specific uses such as oncolytics, thrombolytics, or anticoagulants and as replacements for metabolic deficiencies. Proteolytic enzymes serve as good anti-inflammatory agents. The list of enzymes which have the potential to become important therapeutic agents and its microbial sources are shown in Table 3 and Table 4 respectively. A number of factors severely decrease the potential utility of microbial enzymes once we enter the medical field due to large molecular size of biological catalyst which prevents their distribution within somatic cells, and another reason is the response of immune system of the host cell after injecting the foreign enzyme protein.

**Figure 5:**
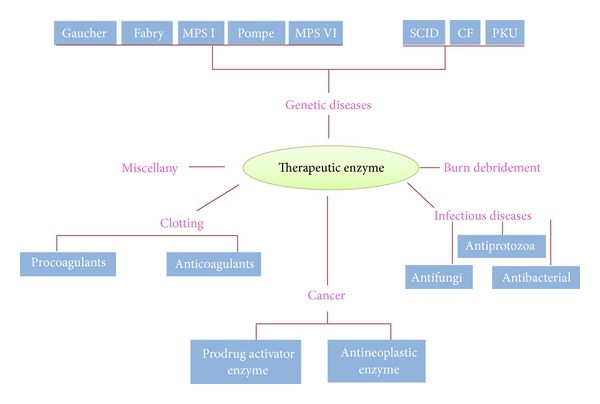
Application of therapeutic enzymes in different disorders and diseases [49–52].

As compared to the industrial use of enzymes, therapeutically useful enzymes are required in relatively less amounts, but the degree of purity and specificity should be generally high. The kinetics of these enzymes are low *K*_*m*_ and high *V*_max⁡_ so that it is maximally efficient even at low concentrations of enzymes and substrates. The sources of such enzymes should be selected with great care to prevent any possibility of undesirable contamination by incompatible material and also to enable ready purification. Therapeutic enzymes are usually marketed as lyophilised pure preparations with biocompatible buffering salts and mannitol diluent. The cost of these enzymes is high but comparable to those of therapeutic agents or treatments. As an example, urokinase is derived from human urine and used to dissolve blood clots. One of the major applications of therapeutic enzymes is in the treatment of cancer and various other diseases as shown in the Figure 5. For the treatment of acute lymphocytic leukaemia asparaginase enzyme has proved to be promising. Its activity depends upon the fact that tumour cells lack aspartate-ammonia ligase activity, which stops the synthesise of nonessential amino acid L-asparagine. Hence, they are extracted from body fluids. The asparaginase does not affect the normal cells which are capable of synthesizing enough for their own requirements, but they decrease the free exogenous concentration, so it causes a state of fatal starvation in the susceptible tumour cells. The enzyme can be administered intravenously and is only effective in reducing asparagine levels within the bloodstream, showing a half-life of about a day (in a dog). This half-life can be increased by 20-fold with use of polyethylene glycol-modified asparaginase.

### 4.1. Treatment of Damaged Tissue

A large number of proteolytic enzymes of plant and bacterial origin have been studied for the removal of dead skin of burns. Various enzymes of higher quality and purity are now in clinical trials. Debrase gel dressing, containing a mixture of several enzymes extracted from pineapple, received clearance in 2002 from the US FDA for a Phase II clinical trial for the treatment of partial-thickness and full-thickness burns. A proteolytic enzyme (VibrilaseTM) obtained from *Vibrio proteolyticus* is found to be effective against denatured proteins such as those found in burned skin. The regeneration of injured spinal cord have been demonstrated using chondroitinases, where this enzyme acts by removing the glial scar and thereby accumulating chondroitin sulfate that stops axon growth [99]. Hyaluronidase has also been found to be a similar hydrolytic activity on chondroitin sulphate and may help in the regeneration of damaged nerve tissue [100].

### 4.2. Treatment of Infectious Diseases

Lysozyme is a naturally occurring antibacterial agent and used in many foods and consumer products, as it is able to breakdown carbohydrate chains in bacterial cell wall. Lysozyme has also been found to have activity against HIV, as RNase A and urinary RNase U present selectively degrade viral RNA [101] showing possibilities for the treatment of HIV infection. Chitinases is another naturally occurring antimicrobial agent. The cell wall of various pathogenic organisms, including fungi, protozoa, and helminths is made up of chitin and is a good target for antimicrobials [102]. The lytic enzyme derived from bacteriophage is used to target the cell walls of *Streptococcus pneumonia*, *Bacillus anthracis,* and *Clostridium perfringens *[103]. The application of lytic bacteriophages can be used for the treatment of several infections and could be useful against new drug-resistant bacterial strains.

### 4.3. Treatment of Cancer

The cancer research has some good instances of the use of enzyme therapeutics. Recent studies have proved that arginine-degrading enzyme (PEGylated arginine deaminase) can inhibit human melanoma and hepatocellular carcinomas [104]. Currently, another PEGylated enzyme, Oncaspar1 (pegaspargase), has shown good results for the treatment of children newly diagnosed with acute lymphoblastic leukemia and are already in use in the clinic. The normal cells are able to synthesize asparagine but the cancerous cells cannot and thus, die in the presence of asparagine degrading enzyme. Asparaginase and PEG-asparaginase are effective adjuncts for standard chemotherapy. Another important feature of oncogenesis is proliferation. It has been proved that the removal of chondroitin sulfate proteoglycans by chondroitinase AC and, to a lesser extent, by chondroitinase B, stops tumor growth, metastasis, and neovascularization [105]. The further application of enzymes as therapeutic agents in cancer is described by antibody-directed enzyme prodrug therapy (ADEPT). A monoclonal antibody carries an enzyme specific to cancer cells where the enzyme activates a prodrug and destroys cancer cells but not normal cells. This approach is being utilized for the discovery and development of cancer therapeutics based on tumor-targeted enzymes that activate prodrugs. The targeted enzyme prodrug therapy (TEPT) platform, involving enzymes with antibody-like targeting domains, will also be used in this effort [106].

## 5. Modern Application of Enzyme: A Biotechnology View

Enzymes are one of the most important biomolecules which has a wide range of applications in industrial as well as biomedical field as describe in Table 5. Today it is one of the most important molecules which are widely used since the ancient human civilization. With the growing population and raising need enzymes seem to be one of the most vital molecules that have a great impact in every sector that may be dairy, industrial, agriculture, or medicine. 

Previously in the 19th up till mid-20th century, the world has seen great industrial expansions which we all know as industrial revolution which has created a steep raise in population and its demand for survival thus creating a great impact in the agricultural, industrial, dairy, and medicinal fields. To meet the raising demand, many scientists had put their great effort to develop many chemical processes to meet the demand, but in later years, the harmful effects of using chemical catalysts to fast up the process have come in front of the mankind. Many chemical transformation processes used in various industries have inherent drawbacks from a commercial and environmental point of view. Nonspecific reactions may result in poor product yields. High temperatures and/or high pressures needed to drive reactions lead to high energy costs and may require large volumes of cooling water downstream. Harsh and hazardous processes involving high temperatures, pressures, acidity, or alkalinity need high capital investment and specially designed equipment and control systems. Unwanted by-products may prove difficult or very costly to produce. High chemicals and energy consumption as well as harmful by-products have a negative impact on the environment. Thus, a need for environment friendly process/biocatalyst came in light which again created a great research innovation in different scientific communities thus leading to a new field called “Biotechnology” where different live organisms were utilized to obtain desirable products in an ecofriendly way. In a number of cases, some or all of these drawbacks can be virtually eliminated by using enzymes. Interestingly enzyme reactions may often be carried out under mild conditions; they are highly specific and involve high reaction rates. Industrial enzymes originate from biological systems which can effectively contribute to sustainable development through being isolated from microorganisms which are fermented using primarily renewable resources. In addition, as only small amounts of enzymes are needed in order to carry out chemical reactions even on an industrial scale, both solid and liquid enzyme preparations take up very little storage space. Mild operating conditions enable uncomplicated and widely available equipment to be used, and enzyme reactions are generally easily controlled. Enzymes also reduce the impact of manufacturing on the environment by reducing the consumption of chemicals, water, and energy and the subsequent generation of waste. Developments in genetic and protein engineering have led to improvements in the stability, economy, specificity, and overall application potential of industrial enzymes. When all the benefits of using enzymes are taken into consideration, it is not surprising that the number of commercial applications of enzymes is increasing every year.

Biotechnology offers an increasing potential for the production of goods to meet various human needs. Enzyme technology are a subfield of biotechnology where new processes had been developed and are still developing to manufacture both bulk and high added value products utilizing enzymes as biocatalysts, in order to meet needs such as food (e.g., bread, cheese, beer, and vinegar), fine chemicals (e.g., amino acids, vitamins), agricultural (growth hormones), and pharmaceuticals (insulin). Enzymes are also used to provide services, as in washing and environmental processes (especially clean-up processes) or for analytical and diagnostic purposes. The driving force in the development of enzyme technology, both in academic research and industry, has been and will continue to the development of new and better products, processes, and services to meet these needs along with the improvement of the processes to produce existing products from new raw materials as biomass. The goal of these approaches is to design innovative products and processes that are not only competitive but also meet criteria of sustainability and economic viability. The concept of sustainability was introduced in the World Commission on Environment and Development (WCED, 1987) with the aim to promote the necessary development that meets the needs of the present and future demand without compromising the ability of future generations to meet their own needs. To determine the sustainability of a process, criteria that evaluate its economic, environmental and social impact must be used [113–115]. A positive effect in all these three fields is required for a sustainable process. Criteria for the quantitative evaluation of the economic and environmental impact are in contrast with the criteria for the social impact, easy to formulate. In order to be economically and environmentally more sustainable than the existing processes, a new process must be designed to reduce not only the consumption of resources (e.g., raw materials, energy, air, and water), waste production, and environmental impact but also to increase the recycling of waste per kilogram of product. Because enzymes are highly specific in the reactions they catalyse, an abundant supply of enzymes must be present in cells to carry out all the different chemical transformations required. Most enzymes help break down large molecules into smaller ones and release energy from their substrates. To date, scientists have identified over 10,000 different enzymes. Because there are so many, a logical method of nomenclature has been developed to ensure that each one can be clearly defined and identified.

Thus, from this table we can get a clear idea that the use of enzymes and bioengineering of them is nowadays very much practised in almost every industry. These biomolecules which are also known as biocatalyst too are now playing a very major role in the modern industrial development that is mainly aimed in economical, high efficiency, and ecofriendly production of different products and by-products. Enzymes are now an important area of studies of different human diseases. 

Like other proteins, enzymes are produced inside cells by ribosomes, which link up amino acids into chains. Although the majority of industrial enzymes are produced by microorganisms, the enzymes are formed in exactly the same way as in human cells. The structure and properties of the enzymes produced by a particular cell are determined by the genetic instructions encoded in the deoxyribonucleic acid (DNA) found in chromosomes of the cell. DNA enables the production of specific enzymes through a code consisting of four bases: adenine (A), guanine (G), cytosine (C), and thymine (T). DNA's characteristic double helix consists of two complementary strands of these bases held together by hydrogen bonds. A always pairs with T, while C always pairs with G. The order in which these bases are assembled in the DNA double helix determines the sequence of amino acids in the enzyme protein molecule. Each fully functional segment of DNA—or gene—determines the structure of a particular protein, with each of the 20 different amino acids being specified by a particular set of three bases. Enzyme engineering sometimes also known as protein engineering is a modern term that is used in the application of modifying an enzyme's structure and thus altering/improving its function by modifying the catalytic activity of isolated enzymes to produce new metabolites [141, 142] or to convert from some certain compounds into others which is called biotransformation. These products will be useful as chemicals, pharmaceuticals, fuel, food, or agricultural additives. Since the tight control of enzyme activity is essential for homeostasis, any malfunction (mutation, overproduction, underproduction, or deletion) of a single critical enzyme can lead to a genetic disease. The importance of enzymes is shown by the fact that a lethal illness can be caused by the malfunction of just one type of enzyme out of the thousands of types present in our bodies. For example, the most common type of enzyme deficiency disorder is phenylketonuria, and a mutation of a single amino acid in the enzyme phenylalanine hydroxylase (which catalyzes the amino acid phenylalanine) results in buildup of phenylalanine and related products. This can lead to mental retardation if the disease is untreated in early childhood [143]. Another example of enzyme deficiency is germline mutations in genes coding for DNA repair enzymes which cause hereditary cancer syndromes such as xeroderma pigmentosum [144]. Defects in these enzymes cause cancer since the body is less able to repair mutations in the genome. This causes a slow accumulation of mutations and results in the development of many types of cancer in the sufferer. Some enzymes are produced in increasing amounts for therapeutic purposes; this applies especially to recombinant enzymes such as factor VIII, tPA, and urokinase that cannot be produced in sufficient amounts from natural sources (blood serum or urine). Another advantage of the recombinant production of these enzymes is that possible contamination with pathogenic human viruses (HIV, herpes) can be avoided. Enzymes are now orally administrated to treat several diseases (e.g., pancreatic insufficiency and lactose intolerance). Since enzymes are proteins themselves, they are potentially subject to inactivation and digestion in the gastrointestinal environment. Therefore a noninvasive imaging assay had been developed to monitor gastrointestinal activity of exogenous enzymes like prolyl endopeptidase as potential adjuvant therapy for celiac disease [145].

## 6. Problems, Safety Concerns, and Possible Future Strategy to Outcome This Problem

Proteins are abundant in nature. Many proteins can cause allergies like pollen, house dust mites, animal dander, and baking flour. Like many other proteins foreign to the human body, enzymes are potential inhalation allergens. The inhalation of even small amounts of foreign protein in the form of dust or aerosols can stimulate the body's immune system to produce specific antibodies. In some individuals, the presence of these specific antibodies can trigger the release of histamine when reexposed to the allergen. This compound can cause symptoms well known to hay fever sufferers such as watery eyes, a runny nose, and a sore throat. When exposure ceases, these symptoms also cease. Enzymes must be inhaled for there to be a risk of causing sensitization that may lead to an allergic reaction. It may be necessary to monitor the working environment in facilities where enzymes are used, especially if large quantities are handled on a daily basis. Monitoring is used to confirm that threshold limit values (TLVs) for airborne enzymes are not being exceeded. In many countries, the TLVs for enzymes are based on the proteolytic enzyme subtilisin and are stated as 0.00006 mg/m^3^ of pure crystalline subtilisin in air [146]. But one industry that has come a long way in the safe handling of enzymes is the detergent industry. The use of encapsulated enzymes, combined with improved industrial hygiene and operating practices, has brought levels of airborne enzyme dust down dramatically in developed countries since the occupational problem of enzyme allergies first came to light in the late 1960s. The trade association AISE has generated a guide to safe handling of enzymes in the detergent industry [147]. It should be emphasized that allergy to enzymes is solely an occupational hazard, and no effects on end consumers using products containing enzymes have ever been reported during more than 35 years of use. In one of the most important reports on the subjects, the National Research Council (NRC), USA, concluded that consumers of enzymatic laundry products did not develop respiratory allergies [148]. Further studies of enzyme allergy over the years have confirmed that enzymatic laundry and dishwashing detergents are safe for consumers to use. The HERA Risk Assessment document gives a comprehensive overview of consumer safety in regards to enzyme application within the household cleaning sector. The safe use of enzymes in food processing has been documented in a recent study by Novozymes and the University Hospital of Odense, Denmark [149].

The application of enzymes in food processing is governed by food laws. Within the EU, large parts of the food laws of individual member states have been harmonized by directives and regulations. For general purposes, the FAO/WHO Joint Expert Committee on Food Additives (JECFA) and the Food Chemicals Codex (FCC) have made guidelines available for the application of enzymes as food additives. AMFEP in Europe and the Enzyme Technical Association (ETA) in the USA work nationally and internationally to harmonize enzyme regulations. AMFEP members ensure that the enzymes used in food processing are obtained from nonpathogenic and nontoxigenic microorganisms, that is, microorganisms that have clean safety records without reported cases of pathogenicity or toxicosis attributed to the species in question. When the production strain contains recombinant DNA, the characteristics and safety record of each of the donor organisms contributing genetic information to the production strain are assessed. The majority of food enzymes are used as processing aids and have no function in the final food. In this case, they do not need to be declared on the label because they are not present in the final food in any significant quantity. A few enzymes are used both as processing aids and as food additives. When used as additives, they must be declared on the food label. Good Manufacturing Practice is used for industrial enzymes for the food industry. The key issues in GMP are microbial control of the microorganism selected for enzyme production, the control and monitoring systems ensuring pure cultures and optimum conditions for enzyme yield during fermentation, and the maintenance of hygienic conditions throughout the recovery and finishing stages.

Commercial enzyme products are usually formulated in aqueous solutions and sold as liquids or processed into nondusting, dry products known as granulates or microgranulates. Both liquid and dry preparations must be formulated with the final application in mind. It is important for both the producer and customer to take into account storage stability requirements such as stability of enzyme activity, microbial stability, physical stability, and the formulation of the enzyme product itself. Enzyme molecules are far too complex to synthesize by purely chemical means, with a very instability of its pure form, and so the only way of making them is to use living organisms. The problem is that the useful enzymes produced by microorganisms in the wild are often expressed in tiny amounts and mixed up with many other enzymes. These microorganisms can also be very difficult to cultivate under industrial conditions, and they may create undesirable by-products. Even there is a possibility of cross-contamination and production of toxins which may be lethal if used.

Genetic engineering is a far more efficient option because the changes are completely controlled. This process basically involves taking the relevant gene from the microorganism that naturally produces a particular enzyme (donor) and inserting it into another microorganism that will produce the enzyme more efficiently (host). The first step is to cleave the DNA of the donor cell into fragments using restriction enzymes. The DNA fragments with the code for the desired enzyme are then placed, with the help of ligases, in a natural vector called a plasmid that can be transferred to the host bacterium or fungus. The DNA added to the host in this way will then divide as the cell divides, leading to a growing colony of cloned cells each containing exact replicas of the gene coding for the enzyme in question. Since the catalytic properties of any enzyme are determined by its three-dimensional structure, which in turn is determined by the linear combination of the constituent amino acids, we can also alter an enzyme's properties by replacing individual amino acids. For example, detergent enzymes can be made more bleach-stable using this type of protein engineering. Bleach-stable protein engineered enzymes have been on the market for a number of years, for example, Novozymes' Everlase. Furthermore, enzymes can be given other useful properties using this technique, for example, improved heat stability, higher activity at low temperatures, and reduced dependency on cofactors such as calcium. New and exciting enzyme applications are likely to bring benefits in other areas like less harm to the environment, greater efficiency, lower cost, lower energy consumption, and the enhancement of a product properties. New enzyme molecules capable of achieving this will no doubt be developed through protein engineering and recombinant DNA techniques. Industrial biotechnology has an important role to play in the way modern foods are processed. New ingredients and alternative solutions to current chemical processes will be the challenge for the enzyme industry. When compared with chemical reactions, the more specific and cleaner technologies made possible by enzyme-catalyzed processes will promote the continued trend towards natural processes in the production of food.

## 7. Conclusion

Enzymes are being known to mankind since the ancient human civilization. The use of enzymes had been done intensively in different fields especially, in ancient brewing and other uses. But since the 18th century it has been technically known to us as enzymes. Many scientists had tried to study the use of enzymes, and from their pioneer work, we have come to know about its power and utility in our daily life. Today different types of enzymes are being manufactured by many big companies and being sold for their important role in different industries like food, dairy, detergent, and chemical as well as for their important lifesaving therapeutically application. Due to advancement of modern biotechnology and protein engineering a new area of enzyme engineering, has evolved which mainly deals with the purification and stability of these important enzymes. Different microbes as well as other model systems are extensively used for the production of these important biomolecules. Since then many microorganisms and their enzymes with unique function have also been discovered by means of extensive screening, and now they are commonly used in different industrial and medical fields. Development of these medically important enzymes has been at least as extensive as those for industrial applications thus reflecting the magnitude of the potential rewards of this sector in the near 

future. Enzymes industry is one among the major industries of the world, and there exists a great market for further improvement in this field. Amylase and lipase are few of these mentionable enzymes that have a wide spectrum role in this sector. Its use is almost done in every industry whether it may be detergent, dairy, food, or medicineThis review especially emphasizes the important wide spectrum role of amylase and lipase in various sectors of industries and also discussed the role of other enzymes in therapeutic field. There is an indeed need of future research in these biomolecules which will later be beneficial for the mankind in their relevance.

## Figures and Tables

**Table 1:** Enzyme classes and types of reactions [14].

Enzyme commission number	Class of enzyme	Reaction profile
EC 1	Oxidoreductases	Oxidation reactions involve the transfer of electrons from one molecule to another. In biological systems we usually see the removal of hydrogen from the substrate. Typical enzymes in this class are called dehydrogenases. For example, alcohol dehydrogenase catalyzes reactions of the type R–CH_2_OH + A R–CHO + H_2_A, where A is an acceptor molecule. If A is oxygen, the relevant enzymes are called oxidases or laccases; if A is hydrogen peroxide, the relevant enzymes are called peroxidases.

EC 2	Transferases	This class of enzymes catalyzes the transfer of groups of atoms from one molecule to another. Aminotransferases or transaminases promote the transfer of an amino group from an amino acid to an alpha-oxoacid.

EC 3	Hydrolases	Hydrolases catalyze hydrolysis, the cleavage of substrates by water. The reactions include the cleavage of peptide bonds in proteins, glycosidic bonds in carbohydrates, and ester bonds in lipids. In general, larger molecules are broken down to smaller fragments by hydrolases.

EC 4	Lyases	Lyases catalyze the addition of groups to double bonds or the formation of double bonds through the removal of groups. Thus bonds are cleaved using a principle different from hydrolysis. Pectate lyases, for example, split the glycosidic linkages by beta-elimination.

EC 5	Isomerases	Isomerases catalyze the transfer of groups from one position to another in the same molecule. In other words, these enzymes change the structure of a substrate by rearranging its atoms.

EC 6	Ligases	Ligases join molecules together with covalent bonds. These enzymes participate in biosynthetic reactions where new groups of bonds are formed. Such reactions require the input of energy in the form of cofactors such as ATP.

**Table 2:** A selection of enzymes used in industrial processes.

Sl no.	Class	Industrial enzymes
1	Oxidoreductases	Catalases Glucose oxidases Laccases

2	Transferases	Fructosyltransferases Glucosyltransferases

3	Hydrolases	Amylases Cellulases Lipases Mannanases Pectinases Phytases Proteases Pullulanases Xylanases

4	Lyases	Pectate lyases Alpha-acetolactate decarboxylases

5	Isomerases	Glucose isomerases Epimerases Mutases Lyases Topoisomerases

6	Ligases	Argininosuccinate Glutathione synthase

**Table 3:** Some important enzymes and their therapeutic importance.

Enzyme	Reaction	Use	Sources	References
Asparaginase	L-Asparagine H_2_O→L-aspartate + NH_3_	Leukaemia	*E. coli *	[82, 83]
Collagenase	Collagen hydrolysis	Skin ulcers	*C. perfringens *	[84]
Glutaminase	L-Glutamine H_2_O→L-glutamate + NH_3_	Leukaemia	*E. coli* SFL-1	[85, 86]
Lysozyme	Bacterial cell wall hydrolysis	Antibiotic	*Homo sapiens *	[87, 88]
Ribonuclease	RNA hydrolysis	Antiviral	Yeast and bacteriophages	[89, 90]
Streptokinase	Plasminogen→plasmin	Blood clots	*Streptococci *sp.	[91]
Trypsin	Protein hydrolysis	Inflammation	*Homosapiens* and* other *vertebrates	[92]
Uricase	Urate + O_2_→ allantoin	Gout	*A. flavus *	[93, 94]
Urokinase	Plasminogen→plasmin	Blood clots	*Bacillus subtilis *	[95, 96]
*β*-Lactamase	*β*-Lactam ring hydrolysis	Antibiotic resistance	*Citrobacter freundii*, *Serratia marcescens*, and *Klebsiella pneumonia *	[97]
Penicillin acylase	Binding the rings of benzylpenicillin (penicillin G) and phenoxymethylpenicillin (penicillin V)	Penicillin production/broad spectrum antibiotic production	*Penicillium *sp.	[98]

**Table 4:** List of some common enzymes found from different species.

Source	Enzyme	Microorganism	References
Fungal	Amylase Glucosidases Proteases Pectinases Glucose oxidase Catalase	*Aspergillus oryzae* *Aspergillus flavus* *Aspergillus niger* *Aspergillus niger * *Penicillium notatum* *Aspergillus niger*	[107–110]

Bacterial	Amylases Proteases Penicillinase	*Bacillus subtilis *	[111]

Yeast	Invertase Lactase	*Saccharomyces cerevisiae* *Saccharomyces fragilis *	[112]

**Table 5:** A broad spectrum idea about using the application of enzymes in different areas.

Types of industries	Enzymes	Use	References
Alcohol/beverage	Amylase, glucanases, proteases, beta-glucanases, arabinoxylans, amyloglucosidase, pullulanases, and acetolactate decarboxylase	Degradation of starch and polycarbonated into simple sugar. Also for degrading complex proteins into sugars thus to increase the fermentation efficiency. Production of low calorie beer	[23, 116–123]

Fruit drinks	Cellulases, pectinases	Clarify fruit juice	[124, 125]

Baby food	Trypsin	Predigest baby foods	[126]

Food processing	Amylase, protease, and papain	Degradation of starch and complex proteins, softening of meat	[127–129]

Dairy	Rennin, lipases, and lactases	Hydrolysing protein, cheese production (Roquefort cheese), and glucose production from lactose	[130–132]

Detergent	Protease, amylase, lipase, cellulases, and mannanase	To remove protein after staining, remove insoluble starch in dish washing, removing oils and fats, and to increase the effectiveness of detergents	[23, 124, 128, 131, 133]

Textile	Amylase, pectinase, cellulases, catalase, and protease	To remove starch size, glue between the fiber core and the waxes, fabric finishing in denims, degrading residual hydrogen peroxide after the bleaching of cotton, wool treatment, and the degumming of raw silk also known as biopolishing	[23, 124, 125, 128, 134]

Paper and pulp	Amylases, xylanases, cellulases, hemicellulose, ligninases, and esterase	Degrade starch to lower viscosity, aiding sizing, deinking, and coating paper. Xylanases reduce bleach required for decolorizing; cellulases and hemicellulase smooth fibers, enhance water drainage, and promote ink removal; lipases reduce pitch and lignin-degrading enzymes remove lignin to soften paper, for esterification	[23, 124, 135]

Animal feedstock	Phytase	Increase total phosphorous content for growth, increase in phytic acid need	[136]

Rubber	Catalase	Generate oxygen from peroxide to convert latex into foam rubber	[128]

Oil and petroleum	Cellulases, ligninases, and mannanase	Formation of ethanol, forming gel breaker in oil drilling	[133, 135]

Biopolymer/plastic	Laccases, peroxidases, lipases, and transglutaminases	Forming cross-links in biopolymers to produce materials in situ by means of polymerization processes	[131, 137]

Pharmaceutical	Nitrile hydratase, D-amino acid oxidase, glutaric acid acylase, penicillin acylase, penicillin G acylase, ammonia lyase, and humulin	Producing water soluble intermediates, semisynthetic antibiotics, intermediate for aspartame, and biosynthetic human insulin	[138, 139]

Molecular biology	Restriction enzymes, DNA ligase, and polymerases	Used to manipulate DNA in genetic engineering, essential for restriction of digestion and the polymerase chain reaction, also important in forensic science	[140]
